# Extrafloral nectar fuels ant life in deserts

**DOI:** 10.1093/aobpla/plu068

**Published:** 2014-11-07

**Authors:** Adriana Aranda-Rickert, Patricia Diez, Brigitte Marazzi

**Affiliations:** 1 Centro Regional de Investigaciones Científicas y Transferencia Tecnológica de La Rioja—CRILAR—(CONICET), Entre Ríos y Mendoza s/n, 5301 Anillaco, La Rioja, Argentina; 2 Facultad de Ciencias Agrarias, Instituto de Botánica del Nordeste—IBONE—(UNNE-CONICET), Sgto. Cabral 2131, 3400 Corrientes, Argentina

**Keywords:** Ant–plant interactions, arid lands, extrafloral nectaries, phenology, plant defence, protective mutualisms

## Abstract

Interactions mediated by extrafloral nectary (EFN)-bearing plants that reward ants with a sweet liquid secretion are well documented in temperate and tropical habitats. However, their distribution and abundance in deserts are poorly known. In this study, we test the predictions that biotic interactions between EFN plants and ants are abundant and common also in arid communities and that EFNs are only functional when new vegetative and reproductive structures are developing. In a seasonal desert of northwestern Argentina, we surveyed the richness and phenology of EFN plants and their associated ants and examined the patterns in ant–plant interaction networks. We found that 25 ant species and 11 EFN-bearing plant species were linked together through 96 pairs of associations. Plants bearing EFNs were abundant, representing ca. 19 % of the species encountered in transects and 24 % of the plant cover. Most ant species sampled (ca. 77 %) fed on EF nectar. Interactions showed a marked seasonal pattern: EFN secretion was directly related to plant phenology and correlated with the time of highest ant ground activity. Our results reveal that EFN-mediated interactions are ecologically relevant components of deserts, and that EFN-bearing plants are crucial for the survival of desert ant communities.

## Abstract (precis)

Many ant–plant associations are mediated by extrafloral nectaries (EFNs): nectar-producing structures not related to pollination and commonly found on leaves and inflorescences. These sweet secretions represent a critical energy resource for many ant species and constitute the basis for protective mutualisms: by providing ants with food, ants protect plants from herbivores. Although EFN-bearing plants occur in a wide range of habitats and climates worldwide, interactions mediated by EFN-bearing plants are poorly documented in deserts. In a seasonal desert of northwestern Argentina, we show that biotic interactions between EFN-bearing plants and ants are ecologically relevant components of deserts, and that EFN-bearing plants are crucial for the survival of desert ant communities.

## Introduction

For many ant species, carbohydrates represent a critical energy resource for colony growth, worker activity and worker survival (Davidson *et al.* 2003; Grover *et al.* 2007; Lach *et al.* 2009; Byk and Del-Claro 2011; Wilder *et al.* 2013; Kay *et al.* 2014). In nature, some of the forms in which carbohydrate-rich liquid food is available for ants are extrafloral (EF) nectar, honeydew from sap-feeding hemipterans and secretions from lepidopteran larvae. These sweet secretions constitute the basis for protective mutualisms: plants provide ants with food, and ants protect plants from herbivores and animals from predators (Heil and McKey 2003). The importance of carbohydrates as an energy resource for ants and the abundance of ants in most terrestrial ecosystems might explain why mutualistic associations between ants and plants or animals that provide such sugar-rich liquids have evolved many times and are widespread in nature (Rico-Gray and Oliveira 2007).

Many ant–plant associations are mediated by extrafloral nectaries (hereafter EFNs): nectar-producing structures not related to pollination and commonly found on leaves and inflorescences (Bentley 1977; Koptur 1992*a*). Extrafloral nectar is an aqueous solution, especially rich in mono- and disaccharides (fructose, glucose and sucrose), but also includes free amino acids (Koptur 2005). Extrafloral nectaries are recorded in at least 3941 species from 108 families, including some ferns (Weber and Keeler 2013). Although EFN-bearing plants (hereafter EFN plants) occur in a wide range of habitats and climates worldwide, most studies on their distribution and abundance focus on temperate and tropical forests and savannah-like habitats (e.g. Oliveira and Leitão-Filho 1987; Koptur 1992*b*; Blüthgen and Reifenrath 2003). In contrast, the relevance of these interactions in arid lands at the community level has largely been overlooked. The relatively few published studies dealing with this topic focus on mutualistic systems involving one or few focal EFN plants in the Sonoran Desert (e.g. Ruffner and Clark 1986; Ness *et al.* 2006; Holland *et al.* 2009; Lanan and Bronstein 2013), and the handful of surveys on arid lands are all from communities in the North American subcontinent (Pemberton 1988; Rico-Gray 1989; Rico-Gray *et al.* 1998). This means that there is no information on EFNs for most of the world's arid lands.

Pemberton (1988) found that EFN plants were abundant in communities of the Colorado and Mojave Deserts (USA), and predicted that EFN-mediated ant–plant interactions should be abundant and common also in other desert communities of the world. The rationale behind his prediction consists of four main points. First, the availability of water and sugar resources is limited in dry climates; therefore, EF nectar may represent a valuable resource for ants in deserts (Ruffner and Clark 1986). Second, the strength of the mutualism and the investment of plants in ant defence should increase under water limitation, because the costs of herbivory are particularly high when resources are scarce (Pringle *et al.* 2013). Third, producing carbon-rich defences should be less costly for plants in relatively nitrogen-limited habitats where C is in excess (Folgarait and Davidson 1994); therefore, EFN plants should be common in sunlight-rich habitats, such as deserts. Finally, EFN plants should be abundant where ants are also abundant (Bentley 1976; Keeler 1981*a*); arid and semiarid habitats are indeed known to harbour a relatively high abundance and diversity of ants (MacKay 1991).

Currently, there is little support for Pemberton's prediction, because the distribution and abundance of EFN plants were only explored in the arid lands of North America. Therefore, in this study, we aimed at filling this gap by investigating the abundance and richness of EFN-mediated ant–plant interactions in the Monte Desert of northwestern Argentina. The Monte Desert covers ∼460 000 km^2^ of the land surface and constitutes the most arid rangeland of Argentina (Abraham *et al.* 2009). Ants are abundant and diverse (Kusnezov 1963), and legumes and cacti, both plant families known for including EFNs (Weber and Keeler 2013), are well represented (Aranda-Rickert 2014). This biome also shows remarkable similarities with the North American deserts of Chihuahua, Mojave and Sonora, with the same climate subtype, the same dominant vegetation and the same combination of biological forms (Solbrig 1976; Mares *et al.* 1985). All these features, along with the lack of studies on the systematic distribution of plants with EFNs and their associated ants, make this biome an ideal field for exploring Pemberton's prediction. Therefore, we addressed the following questions: how abundant are EFN plants in the Monte Desert communities? Which species of ants are involved in EFN-mediated interactions and what proportion of the ant community do they represent? Is there any seasonal variation in the intensity of interactions?

Based on the ‘optimal defence theory’, which states that defences should be deployed among plant parts in direct proportion to their value and likelihood of attack (Rhoades 1979; Holland *et al.* 2009), and assuming that reproductive and young vegetative plant parts are particularly vulnerable to herbivory, we predict that EFNs are only functional when new vegetative and reproductive structures are developing. Therefore, we expect that nectar secretion activity correlates with plant phenology and abundance of EFN-visiting ants. Furthermore, we analysed the structure of ant–plant networks in two different communities of the Monte Desert, and discussed the observed patterns of co-occurrence and dominance of ant species on EFN plants. The results of this study will increase our understanding of how EFN resources may influence ant species composition, ant abundance and interactions at the community scale and how important are EFN-mediated interactions with ants for the ecology of desert communities.

## Methods

### Study sites

We conducted this study at two sites near Anillaco (28°48′S, 66°56′W), La Rioja Province, northwestern Argentina. The study area is located in the northern portion of the Monte Desert, on the east slope of the Sierras de Velasco mountain range. The climate is arid and seasonally marked. Average annual temperature is 16.6 °C, and average annual precipitation is 272 mm, falling mainly during the December–March summer wet season (Anillaco Meteorological Station, data from 1999 to 2012). The two selected sites are 15 km apart from each other and relatively undisturbed. The ‘Jarillal’ site (1100 m a.s.l., above sea level), located on a gentle slope and characterized by fine-textured soils, is an open shrubland dominated by *Larrea cuneifolia* (‘jarilla’) and *Bulnesia retama* (Zygophyllaceae). The ‘Piedmont’ site (1400 m a.s.l.) is located on a mid-slope (3–4 % steepness), where the soil texture is coarse and rocky deposits are frequent. Its shrubby vegetation is dominated by *Flourensia fiebrigii* (Asteraceae) and *L. cuneifolia.* Both sites are rich in perennial Cactaceae and Fabaceae, which are characteristic elements also elsewhere in the Monte Desert (Aranda-Rickert 2014).

### Plant survey and EFN detection

We surveyed plants in February 2013. This month coincides with the period of maximum number of plant species after the summer rains, including ephemeral and annual species (otherwise absent), and thus provides more precise estimates of the abundance and richness of EFN plants relative to the total vegetation. Within each site, we used a metre tape to define three 100-m-long transects that were randomly positioned and at least 100 m apart from one another. We estimated the percent aerial cover of plants with EFNs through the point-intercept method (Floyd and Anderson 1987). Sampling points were established every 1 m along each transect, for a total of 100 points/transect and 300 points/site. At each point we dropped sample pins (1 m in height), and all plant species touching the pin were recorded. Percent cover of EFN plants per site was estimated by dividing the total number of pin drops touched by an EFN plant by the total number of points per site. We also estimated the percentage of EFN plant species by recording all plant species intercepted by the line transect. Plant species were recorded only once and we did not distinguish between live and dead leaves and stems.

Each encountered plant species was visually examined in detail to detect the presence of EFNs on vegetative and reproductive parts. We also searched for EFNs in plant species not included within the sampled transects. We guided our inspection using online taxonomic lists of families and genera bearing EFNs (Keeler 2008). Presence of EFNs was confirmed by collecting the plant parts and examining them in the lab. The observation of stereotypical nectar-gathering behaviour of ants or other nectar feeders (i.e. immobile and with mouthparts in contact with nectar-secreting tissues for periods of up to several minutes) also served as a cue for detecting the presence and location of EFNs (Rico-Gray 1993). This was particularly helpful when no previous report of EFN presence existed for the genus or species and/or when no macroscopic EFN structures could be observed. In these last cases, we also used glucose test strips (Diabur-Test 5000; Roche Diagnostics GmbH, Germany) to confirm sugar secretion (Díaz-Castelazo *et al.* 2005). We considered a plant species as an EFN plant when EF nectar was observed at least in one of its phenological stages.

Anatomical analyses were necessary in one species, *Senna rigida*. In this leafless plant, it has been suggested that EFNs could occur in the inflorescence, as in other species of a larger clade *S. rigida* belongs to (Marazzi *et al.* 2013). Indeed, we observed ant-feeding behaviours that could indicate EFNs' presence at the base of peduncles and pedicels of lateral inflorescences. Furthermore, in some cases, we observed droplets accumulating at the abscission site of pedicels and peduncles. Therefore, to confirm the presence of nectar-secreting structures, we analysed the intersection between peduncles and pedicels and their respective axes (and respective bracts) using standard procedures for microtome sectioning (as in Gonzalez and Ocantos 2006). Two FAA-fixed samples of each collected plant were embedded in paraffin (Johansen 1940) for transversal and longitudinal sections, respectively, and cut at 12.5 µm using a Microm rotary microtome (Thermo Fisher Scientific, Inc., USA). Sections were mounted on glass slides and stained with Safranin and Astra Blue (Luque *et al.* 1996) and then inspected and photographed using a Leica DM LB2 light microscope with an embedded ICC50 digital camera (Leica Microsystems CMS GmbH, Germany).

### Ant survey

To determine the identity and abundance of ants feeding on EFN plants, we conducted a 2-min survey on every plant along the three 100-m-long transects used for the plant survey (see previous section). During each survey we recorded the ant species, the number of individuals per species and the location and type of EFNs visited by the ants. When the species could not be identified by the naked eye, as was mainly the case for tiny ants, a few individuals were collected for identification in the lab using a stereomicroscope and regional keys (Kusnezov 1978).

We used pitfall traps to determine the diversity and abundance of ground-dwelling ants. Pitfall traps consisted of 4-cm diameter plastic cups (30 ml volume) partially filled with propylene glycol. Pitfall traps were buried flush with the soil surface and left for 72 h. Traps were placed every 10 m along the survey transects for a total of 10 traps per transect and 30 per site. All traps were operated simultaneously at the two sites. We additionally hand-collected samples of arboreal ant species, usually not caught in pitfall traps. Data from pitfall traps and hand collecting were pooled for each site to estimate the proportion of the local ant species assemblage that forages on EFN plants. We considered as nectar consumers those ant species that were observed feeding on EFNs during the surveys.

### Seasonal variation

To investigate seasonal variation in the interactions, the survey of ants feeding on EFNs and that of ants caught with pitfall traps were taken simultaneously and repeated on the same transects eight times during 1 year (from November 2012 to November 2013) covering both the warm (October–March) and cold seasons (May–August). In order to establish a seasonal pattern in the supply of EFN resources, we recorded the plant phenological stage (vegetative growth, flowering and fruiting), the activity of EFNs (secreting or not secreting) and also the presence of ants feeding on the EFNs. All surveys were done during the time of the day with main ant activity: 1100–1500 h during the cold months and 1800–2000 h during the warm months.

We used two separate two-way analysis of variance to analyse seasonal changes in the frequency of interactions and in the abundance of nectar-consuming ant species. The response variables were the abundance of ants on EFN plants (number of workers observed interacting with an EFN plant) and the abundance of nectar-consumer ants in pitfall traps (number of ground-dwelling ant workers). Sites (Jarillal and Piedmont) and months were the independent factors. Data were square-root transformed prior to analysis to meet parametric assumptions. Post-hoc Tukey honestly significant difference tests were performed to explore in which month the differences were found. We analysed the relationship between the abundance of ants on EFN plants and the abundance of nectar-consumer ants in pitfall traps with a Spearman's rank correlation (since our data is non-normal). The level of significance for all statistical analyses was at a *P*-value of 0.05. All statistical analyses (including the network analyses below) were conducted in R v.3.1.1 (R Development Core Team 2014).

### Ant–EFN interactions

For each site, we pooled the data collected across all censuses to construct a plant–ant weighted (quantitative) interaction network. Plants and ants are linked nodes when an interaction is observed between them, and each link has a specific weight depending on the interaction frequency. These networks were represented by a quantitative interaction matrix *p* × *a*, where *p* is the number of plant species, *a* is the number of ant species and the value in each matrix cell, *n_ij_* is the number of interactions, measured as the number of times each ant species *j* was recorded on each plant species *i* **[see Supporting Information]**. We used the network- and group-level functions in the R-package ‘bipartite’ (Dormann *et al.* 2008) to calculate the mean number of links per ant and per plant species (the sum of links for each species averaged over all species in that level) and the network level of specialization (specialization index H_2_′). This index is derived from the Shannon diversity of network links and is based on the deviation of a species' realized number of interactions and that expected from each species' total number of interactions (0 = no specialization, 1 = complete specialization). The level of generalization of a given species was defined as equal to the number of links (i.e. extreme specialists are species that interact only with one partner and extreme generalists are those that interact with all possible partners).

## Results

### Plant survey

Of the 63 species of plants encountered in transects in the Jarillal and Piedmont sites, we found 11 EFN-bearing plant species (17.46 % of total species sampled). The Piedmont had a greater number of species (46) than the Jarillal site (34), but less EFN species (17.4 %, eight species vs. 20.6 %, seven species, respectively). The percent cover of EFN plants was higher in the Piedmont (30.6 %, 92/300 points) than in the Jarillal site (17 %, 51/300).

**Figure 1.**
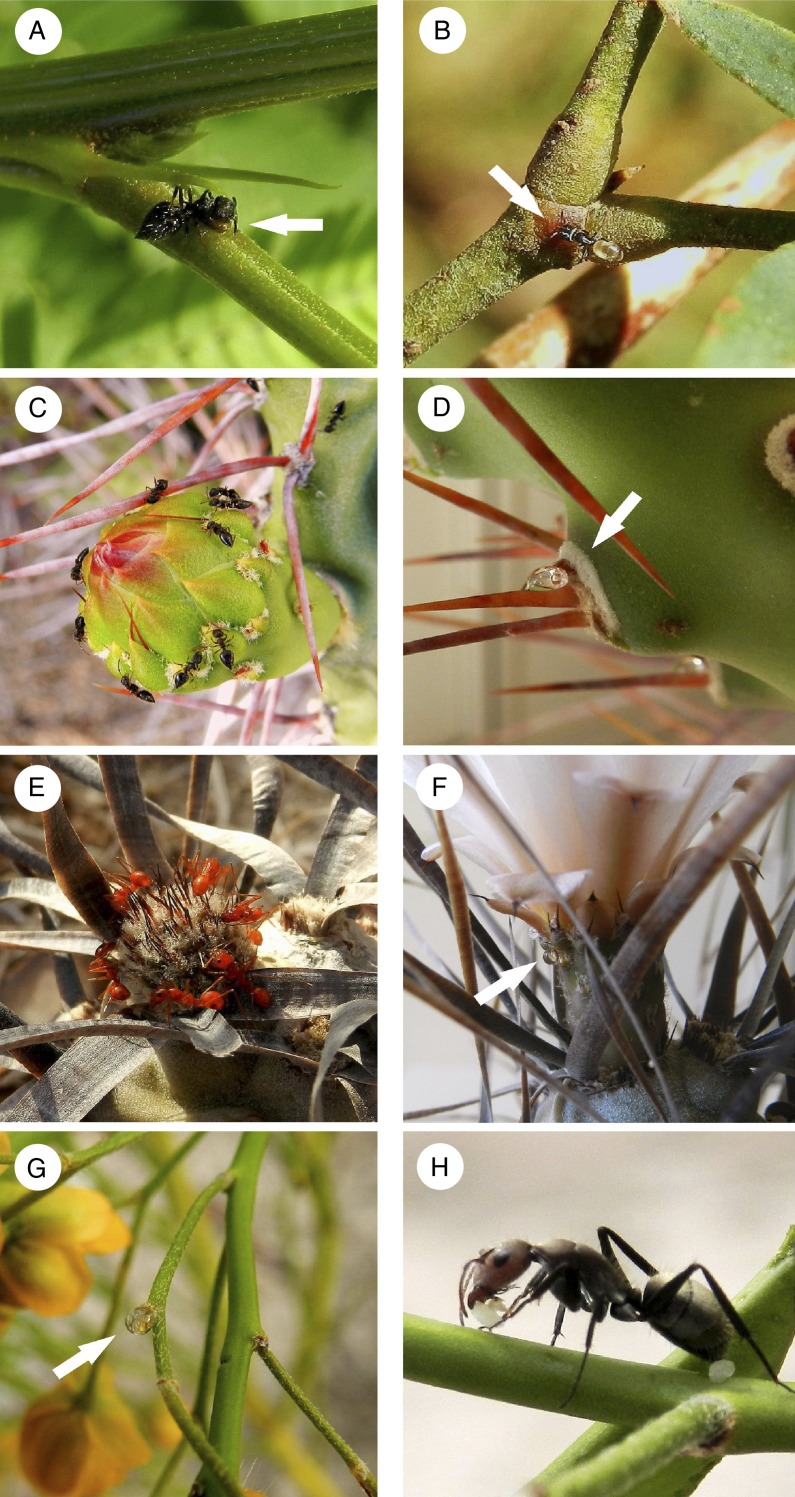
Diversity of the interactions between EFN plants and ants in the Monte Desert: (A) *Cr. quadriformis* ant feeding on *A. visco* EFN, (B) *F. albiventris* ant feeding on *P. flexuosa* EFN, (C) *Cr. quadriformis* ants on a flower bud of *O. sulphurea*, (D) droplet of EFN secreted from the areoles of young cladodes of *O. sulphurea*, (E) *D. planidens* ants on a cladode bud of *T. articulatus*, (F) droplet of EF nectar secreted from areoles of the flower cup of *T. articulatus*, (G) droplet of EF nectar secreted from the scar of an abscised flower of *S. rigida*, and (H) *C. blandus* ant predating on an unidentified insect larvae on *S. rigida.* Arrows indicate the site of EFNs.

**Figure 2.**
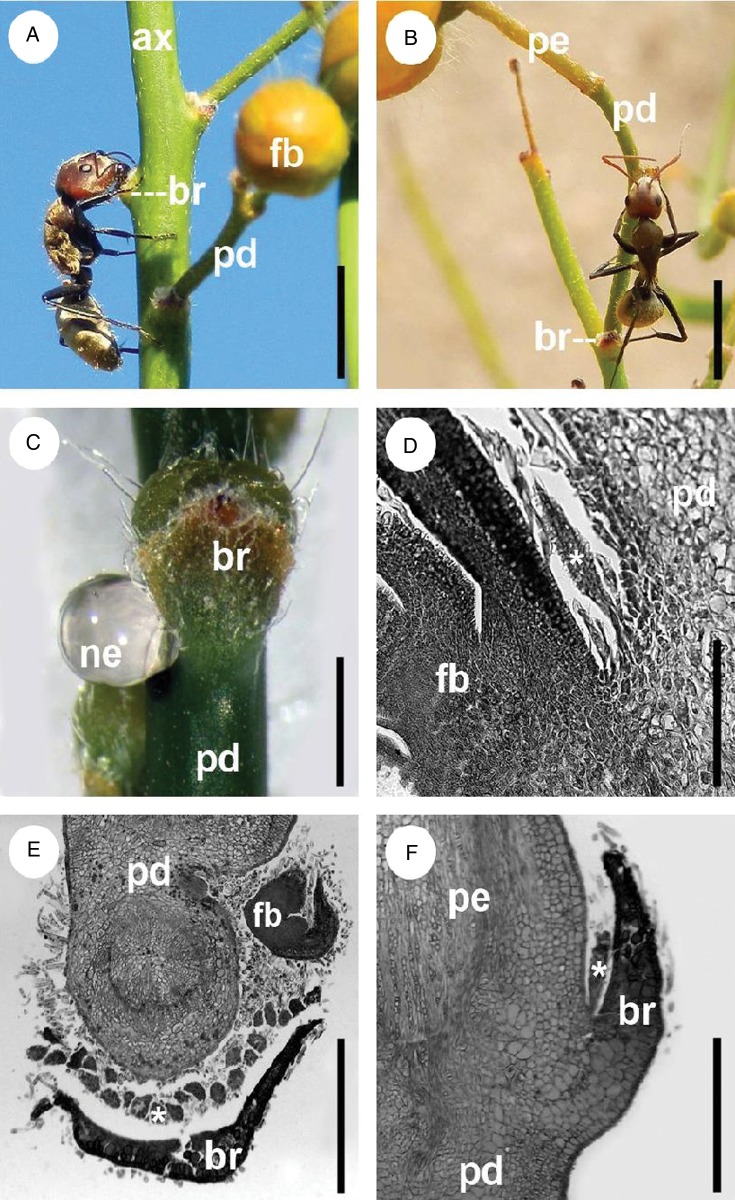
Extrafloral-nectar secreting structures in *S. rigida*. (A, B) location of secretory structures at abscission site of peduncles as indicated by feeding behaviour of *C. blandus*; (A) main inflorescence axis, (B) lateral inflorescence; (C) nectar droplet on an ant-excluded inflorescence; (D–F) microtome sections identifying the secretory structures (indicated by asterisks) at the base of peduncles and pedicels, (D, F) longitudinal sections, (E) transversal section. ax, axis of inflorescence; br, bract; fb, floral bud; pd, peduncle; pe, pedicel. Approximate scale bars: (A, B) 5 mm; (C) 2 mm; (D) 0.25 mm; (E, F) 1 mm.

The EFN species belong to three angiosperm families and six genera (seven species from Fabaceae, three from Cactaceae and one from Bromeliaceae; all perennials; Table 1). Except for *Deuterocohnia longipetala* (Bromeliaceae), to our knowledge none of these species were previously reported to interact with ants. Extrafloral nectaries are reported for the first time in four of these species, *Opuntia sulphurea*, *Tephrocactus alexenderi*, *T. articulatus* and *S. rigida* (it is also the first report for the genus *Tephrocactus*). In the mimosoid legumes (*Acacia* and *Prosopis* species), EFNs are conspicuous and gland-like and are located on vegetative plant parts (Fig. 1A and B), whereas they are rather inconspicuous and located on reproductive structures in *D. longipetala* and *S. rigida* (Fig. 1G). In *D. longipetala*, EFNs are morphologically cryptic or non-individualized (*sensu* Marazzi *et al.* 2013), whereas in *S. rigida*, they consist of multicellular glandular trichome-like structures concentrated between the peduncle or pedicel base and the respective subtending bract (Fig. 2). The three cacti (*Opuntia* and *Tephrocactus* species) bear EFNs in the form of modified spines on the areoles of new sterile cladodes and of cladodes supporting reproductive structures, as well as on the dorsal side of floral bracts (Fig. 1C–F). We observed no EFNs in annual herbaceous species or grasses and found no species with domatia or food bodies.

**Table 1.** List of EFN-plant species and EFN-consumer ant species in the Monte Desert. Plant species are based on a systematic survey in two sites and three 100 m transects site^−1^. Ant species and number of visited EFN-plant species are based on eight surveys of 2 min EFN plant^−1^ between November 2012 and November 2013. Life form: sh, shrub (incl. trees <5 m); tr, tree; he, perennial herb. *First report of EFNs on the plant species; ^†^first report of EFNs on the plant genus. Superscript numbers indicate references from previous reports of EFNs for species: ^1^Galetto and Bernardello (1992); ^2^Vilela and Palacios (1997); ^3^Cialdella (1984).

Plant family/species	Life-form	Location of nectary	Ant family/species	No. of plant species visited
Bromeliaceae			Dolichoderinae	
*Deuterocohnia longipetala*^1^	He	External part of tepals	*Dorymyrmex breviscapis*	2
			*Dorymyrmex ensifer*	1
			*Dorymyrmex exsanguis*	6
Cactaceae			*Dorymyrmex planidens*	5
Opuntia sulphurea*	Sh	Areoles of developing cladodes, flowers and developing fruits	*Dorymyrmex spurius*	5
			*Dorymyrmex wolffhuegeli*	2
			*Dorymyrmex* sp.1	4
Tephrocactus alexenderi*^†^	Sh		*Forelius albiventris*	6
			*Forelius chalybaeus*	2
			*Forelius rufus*	1
Tephrocactus articulatus*^†^	Sh		Formicinae	
			*Brachymyrmex patagonicus*	7
			*Camponotus blandus*	11
Fabaceae			*Camponotus mus*	11
*Acacia aroma*^3^	Sh	Petiole	*Camponotus punctulatus*	6
			*Camponotus substitutus*	4
			Myrmicinae	
*Acacia gilliesii*^3^	Sh	Petiole, rachis	*Cephalotes bruchi*	2
			*Cephalotes liogaster*	2
*Acacia visco*^3^	Tr	Petiole	*Crematogaster quadriformis*	6
			*Crematogaster rochai*	2
*Prosopis chilensis*^2^	Tr	Petiole, rachis	*Pheidole bergi*	2
			*Pheidole triconstricta* l	1
			*Solenopsis* sp. 1	2
*Prosopis flexuosa*^2^	Tr	Petiole, rachis	*Solenopsis* sp. 2	2
*Prosopis torquata*^2^	Sh	Petiole	Pseudomyrmycinae	
			*Pseudomyrmex denticollis*	3
Senna rigida*	Sh	Base of pedicel, abscission site of pedicels and peduncule of lateral inflorescences	*Pseudomyrmex* sp. 1	1
			Total interactions	96

### Ant survey

A total of 34 ant species, 30 at the Piedmont and 23 at the Jarillal site, were collected combining the pitfall traps and the hand-collecting data. All ants were native. At least 25 species in three subfamilies (Myrmicinae, Dolichoderinae and Formicinae) foraged on the 11 EFN plants, forming 96 ant-plant associations (Table 1). Ants foraging on EFNs represented 76.6 and 78.2 % of the epigeal (i.e. ground-dwelling and arboreal) ant assemblage at the Piedmont and Jarillal sites, respectively. Non-EFN-consumer ants included specialized granivores, fungus growers, scavengers and specialized predators **[see Supporting Information]**.

### Seasonal variation

**Figure 3.**
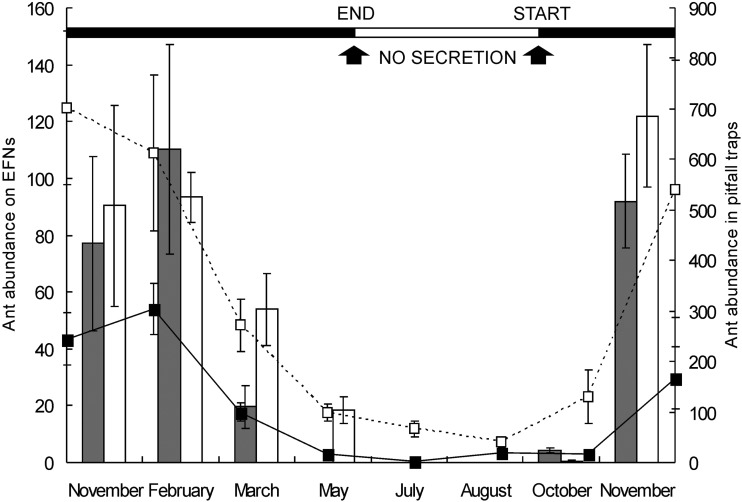
Seasonal variation in the abundance (number) of ants feeding on EFN plants (bars) and the abundance of nectar-consumer ants collected in pitfall traps (lines) in the Jarillal (white bars, open squares) and in the Piedmont site (grey bars, full squares). Data are means (±SE) of three 100 m transects site^−1^, 2 min census EFN plant^−1^ (for ant abundance on EFNs) and 10 pitfall traps transect^−1^ (for ant abundance in pitfall traps). The line above shows the phenology of EFN secretion.

Both the abundance of nectar-consumer ants in pitfall traps and the abundance of ants on EFN plants significantly differed between seasons (*F*_7,32_ = 9.23, *P* < 0.001 and *F*_7,32_ = 15.01, *P* < 0.001, respectively). The abundance of ants in pitfall traps was higher in the Jarillal than in the Piedmont site (mean ± SD: 308.29 ± 311.69 vs. 109.16 ± 122.14, *F*_1,32_ = 19.95, *P* < 0.001). This difference was mainly due to the high abundance of *Dorymyrmex planidens* workers found only in the Jarillal site, contributing to 65.48 % of the total individuals from all species at that site. No significant differences were found in the abundance of ants on EFNs between sites (*F*_1,32_ = 1.17, *P* = 0.28). During the cold and dry months (May to August), interactions were almost absent until the temperatures increased and rains began (early to mid-November). Peak periods of interactions occurred between November and February. This pattern correlated with peak EFN activity (nectar secretion) and peak abundance of nectar-consumer ants in pitfall traps (Fig. 3). The abundance of ants on EFN plants correlated positively with the abundance of nectar-consumer ants in pitfall traps (Spearman's rank correlation: *r*_s_ = 0.79, *P* < 0.001; *n* = 48).

### Patterns of ant–EFN interactions

**Figure 4.**
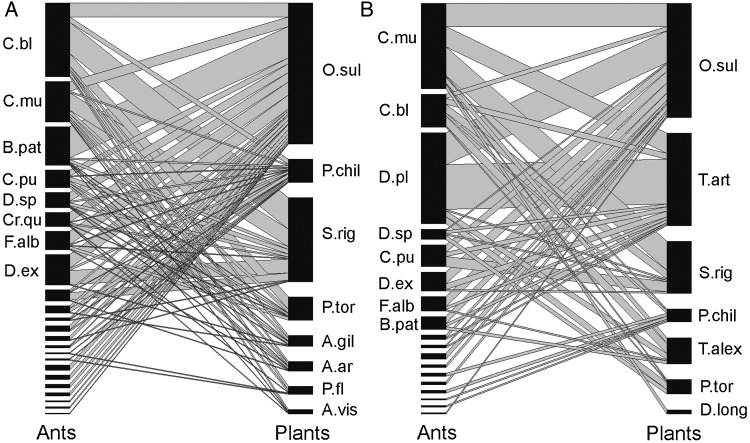
Bipartite graphs showing the interaction network between EFN-ant consumers and EFN plants for the Piedmont (A) and the Jarillal (B) site. Interacting species are linked by lines, and are ordered from top to down by decreasing the number of links. The widths of the links are scaled in relation to the number of interactions between each pair of species and the length of the bars to the total number of interactions for each species (number of visits during eight surveys of 2 min EFN plant^−1^ between November 2012 and November 2013). Names of plant and ant species correspond to the list given in Table 1 (only names of the most frequent ant species are shown).

Networks inferred for each site differed in the number of plant and ant species involved as well as in the number of interactions, yet they shared 4 plant and 16 ant species (Table 2, Fig. 4). Both networks were asymmetric, with more ant than plant species, and little specialization. The plants were more generalist (mean number of links considering the two sites: 12.45, average of the two sites) than were the ants (mean number of links: 5.35). There were no specialist–specialist interactions.

**Table 2.** Network properties of the ant–EFN plant networks at the Jarillal and Piedmont sites.

Network metrics	Piedmont	Jarillal
Number of plant species	8	7
Number of ant species	23	18
Number of links (qualitative data)	77	46
Number of interactions (quantitative data)	417	219
Mean number of links for plant species	15.1	9.8
Mean number of links for ant species	5.9	4.8
Degree of specialization (H_2_′)	0.18	0.21

*Opuntia sulphurea* was the most visited plant species at both sites (in terms of ant species diversity and proportion of total ant visits). It was visited by 21 ant species in the Piedmont (46.28 % of visits) and 14 in the Jarillal site (36.07 %). S*enna rigida* was the second-most visited in the Piedmont (11 ant species, 27.57 % of visits) and the third-most in the Jarillal site (six species, 16.43 %). *Tephrocactus articulatus* was present only at the Jarillal site, where it was the second-most visited (10 species, 29.20 %). The remaining plant species were visited by at least four ant species, except for *D. longipetala*, visited by only one species.

The two sites differed in the ant species representing the most frequent visitor of EFN plants. In the Piedmont site, *Camponotus blandus* was the most frequent (eight plant species, 24.20 % of the visits), followed by *Camponotus mus* (eight species, 13.18 %) and *Brachymyrmex patagonicus* (seven species, 12.71 %). In the Jarillal site, *D. planidens* was the most frequent ant (five species, 28.76 %), followed by *C. mus* (seven species, 26.9 %). *Camponotus mus* and *C. blandus* were the most generalist ants (i.e. with the most links to plants) at both sites. The arboreal *Cephalotes* species were the most specialists, foraging only on EFNs of *Prosopis flexuosa* and *P. chilensis. Dorymyrmex* was the genus with most species (seven) involved in EFN-mediated interactions.

Co-occurrences between different ant species on the same EFN plant were common, but largely determined by dominance hierarchies of the ant assemblage. The dominant species (*Camponotus*, *Crematogaster*, *Solenopsis* and *D. planidens* species) were never found foraging on the same plant but they co-occurred with less aggressive species (*B. patagonicus*, *Dorymyrmex, Forelius*, *Cephalotes* and *Pseudomyrmex* species), though they did not share nectaries. Most ant species nest on the ground, except for *Cephalotes* species, which typically dwell in arboreal nests, and *Camponotus* species, which sometimes nest in living or dead wood. The dominant ant species (*Crematogaster*, *Camponotus* and *D. planidens* species) commonly nested near (<2 m), or in the root system (as in *O. sulphurea* and *T. articulatus*) of the EFN plants they tended.

## Discussion

Harsh environmental conditions, such as those of deserts, are expected to favour food-reward-based mutualistic interactions between species (Thrall *et al.* 2007; Pringle *et al.* 2013). In this study on the Monte Desert of northwestern Argentina, we provide empirical support for the prediction that EFN-mediated interactions with ants are indeed relatively abundant and common in desert communities and crucial for the maintenance of desert ant communities.

### Abundance and richness of EFN-mediated ant–plant interactions in the Monte Desert

Deserts can be considered relatively rich in EFN plants. The number of EFN species in our study on the Monte Desert is similar to that of the North American deserts surveyed by Pemberton (1988; 11 species). Although this number appears to be relatively low, especially if compared with tropical and savannah-like habitats, EFN plants are actually abundant in deserts: one of every four plants in the Monte Desert and in the desert washes of the Colorado and Mojave deserts (Pemberton 1988) bears EFNs. Their abundance is also comparable with those reported for communities long known for their richness of EFN plants, such as the Brazilian cerrado and tropical rainforests (see Table 3).

**Table 3.** Abundance of EFN plants in different habitats worldwide**.** Species and/or individuals (cover) percentages are based on local field surveys recorded on transects or plots. *Only trees, ^†^Only woody species.

Habitat type and location	Vegetation type	Cover (%)	Species (%)	Reference
*Temperate*				
Northern California, USA	Grassland, forest, chaparral	0	0	Keeler (1981*b*)
Southern Florida, USA	Savanna	2.5	12	Koptur (1992*b*)
	Pine forest	34	27	
	Hammock	23	22	
Andros Island, Bahamas	Pine forest	19	28	Koptur *et al.* (2010)
*Desert*				
Colorado and Mojave Deserts, USA	Creosote bush scrub	23.9–27.7	–	Pemberton (1988)
	Desert wash	0.07–6.6		
Northern Monte Desert, Argentina	Open shrubland	17	20.6	This study
	Piedmont	30.6	17.4	
*Tropical and subtropical*				
Cerrado, Brazil	Savanna*	7.6–31.2	15.4–25.5	Oliveira and Leitão-Filho (1987); Oliveira and Oliveira-Filho (1991)
Northern Queensland, Australia	Rainforest^†^	14.4	16.9	Blüthgen and Reifenrath (2003)
Amazon, Brazil	Rainforest^†^	19.1–42.6	17.6–18.5	Morellato and Oliveira (1991)
	Savanna^†^	50	53.3	
West Malaysia	Rainforest^†^	19.3	12.3	Fiala and Linsenmair (1995)
Barro Colorado Island, Panama	Rainforest^†^	–	14–34	Schupp and Feener (1991)
Veracruz, Mexico	Coastal communities	–	14.8	Díaz-Castelazo *et al.* (2004)

Most desert EFN plants were legumes and cacti, both representing typical elements of arid lands in the Americas, and for which the occurrence of EFNs is relatively well documented (Weber and Keeler 2013). Although some of the species of this study have been reported elsewhere as bearing EFNs, their interactions with ants have previously not been described. Furthermore, *Tephrocactus* is a new genus record for EFN presence in Cactaceae. Also, EFNs were previously thought to be absent in *S. rigida* due to the lack of leaves in this and closely related species; therefore, our findings support the view that EFNs in *Senna* are among the most diverse in plants (see Marazzi *et al.* 2013).

Ant abundance has been suggested as an important factor in the distribution and abundance of EFN plants (Keeler 1981*a*). With 34 species, the Monte Desert is similar in ant abundance and diversity to the North American deserts (32 species in the Chihuahuan Desert; Rojas and Fragoso 2000; 39 species in the Sonoran Desert; Bestelmeyer and Schooley 1999; and 26 species in the Tehuacan Valley of Mexico; Rios-Casanova *et al.* 2006).

The guild of ants feeding on EF nectar in the Monte Desert can represent up to 78 % of the epigeal ant community. Although ant species that feed on EF nectar are also partly carnivores or scavengers, for some ant species, honeydew and EF nectar indeed contribute to most of their diet (Fewell *et al.* 1992; Del-Claro *et al.* 2002). Extrafloral nectar is like an energy drink for ants, because it is a source of carbohydrates and amino acids (Davidson 1997; Petanidou *et al.* 2006), and water represents an extremely valuable resource especially in deserts (Ruffner and Clark 1986). Therefore, desert EFN plants and the availability of EF nectar are not only critical to the survival of Monte Desert ant communities, but also contribute to explain ant richness in this desert.

Extrafloral nectar is also a reliable resource in the Monte Desert, since it is available when ants need it most. The Monte Desert is seasonal, and the activity of EFNs is restricted to its spring–summer season, which in turn correlates with the period of highest ant ground activity and EFN foraging activity. From early November to February, the mean number of workers on EFNs was 10 times higher, and the mean number of ground-dwelling nectarivorous ants was nearly six times higher than in the other months of the year. The seasonal pattern of EFN secretion also appears to reflect a temporal distribution of defence investment by EFN plants as predicted by the ‘optimal defence theory’ (Rhoades 1979; Holland *et al.* 2009). Our results indeed show that desert EFNs are functional only when new vegetative and reproductive structures are developing, therefore increasing ant visitation at a time when these structures are most vulnerable to herbivore damage.

### Patterns of ant–EFN plant interactions

The EFN plants were visited by a dynamic and opportunistic ant assemblage. The interaction networks between Monte Desert EFN plants and ants were little specialized (no specialist–specialist interactions) and similar to other nectar-consumer ant communities elsewhere (Guimarães *et al.* 2006; Blüthgen *et al.* 2007; Chamberlain *et al.* 2010). Ant species were 2.5–2.8 times more numerous than the EFN-plant species they visited. Only ants of the genus *Cephalotes,* which commonly nest in cavities of trees and feed primarily on plant and insect exudates (Powell 2008), displayed some degree of specialization, interacting exclusively with *Prosopis* trees.

Co-occurrence of different ant species on the same desert EFN plant was common. We found up to five co-occurring ant species, one of which was a behavioural dominant ant while the others were less dominant or subordinate. Dominant ant species mutually excluded each other from the EFN plant they tended. For example, *C. mus* and *C. blandus*, members of a genus reported as abundant and frequent EFN visitor in various habitats (e.g. Oliveira *et al.* 1999; Díaz-Castelazo *et al.* 2004; Dáttilo *et al.* 2014), were the most common EFN consumers in this study. Both *C. blandus* and *C. mus* are large (7.5–13 mm) and behavioural dominant (Aranda-Rickert and Fracchia 2011) and never occur simultaneously on EFN plants with other aggressive ants, such as *Crematogaster quadriformis*, *D. planidens* and congeneric species.

The most generalist and most frequently visited plant was *O. sulphurea*. This might be explained by the fact that this cactus is abundant in the Monte Desert, consistently secretes EF nectar during the entire spring-summer season and is relatively small, allowing ants to easily access its EFNs found on all the areoles of new vegetative and reproductive structures. In contrast, less visited plants, such as legumes, secrete EF nectar only during the time of inflorescence (e.g. *S. rigida*) or leaf development (e.g. *Prosopis* and *Acacia* spp.), and are relatively larger, requiring ants to navigate from the plants' base up and along the stems and branches to reach the EFNs. Interestingly, all studied *O. sulphurea* plants were consistently visited by the same, and only one, dominant ant species, which nested nearby on the ground or in the root system of the cactus. The assemblage of hierarchically inferior ant species that co-occurred with these dominant ants was temporally variable across cactus individuals, meaning that the subordinate ant species did not show fidelity for a given individual. Therefore, our results suggest that, by providing a predictable and stable resource in the form of EF nectar, *O. sulphurea* shapes the distribution of dominant ant communities. The next step would be to evaluate more accurately whether it plays a key role in ant–plant interactions in the Monte Desert.

## Conclusions

By providing a constant resource to ‘fuel’ the growth of ant populations, mutualisms between ants and EFN plants not only contribute to the structuring of ant communities (Guimarães *et al.* 2007; Díaz-Castelazo *et al.* 2010, 2013), but also shape an array of other arthropods' communities via multitrophic interactions (Davidson *et al.* 2003; Heil and McKey 2003). Our study suggests that this is the case in desert ant communities. Moreover, ant effects on EFN-plants have been shown to be routinely positive and rarely neutral (Chamberlain and Holland 2009), making EFN-mediated ant–plant interactions fundamental to desert plants, in particular, and to ecosystem functioning, in general. The Monte Desert and other arid environments of northwestern Argentina are under risk of desertification (Villagra *et al.* 2009) and, along with the rest of South American and North American deserts, are predicted to be especially affected by global warming (IPCC 2013). Global warming has, for instance, been identified as one of the greatest threats to plant biotic interactions in the Sonoran Desert (B. Marazzi *et al.*, unpubl. data). Knowledge of the web of relationships that structure desert communities is crucial to make comparisons with future surveys of ant–plant mutualisms and to guide conservation and restoration efforts aimed at preserving the biodiversity of deserts.

## Contributions by the Authors

A.A.-R. conceived and designed the study; A.A.-R., P.D. and B.M. performed the field work and analysed the data, A.A.-R., P.D. and B.M. contributed to writing the manuscript.

## Conflicts of Interest Statement

None declared.

## Supplementary Material

Additional Information

## Supporting Information

The following Supporting Information is available in the online version of this article –

**Table S1.** Weighted interaction matrix (number of visits) for the Jarillal site.

**Table S2.** Weighted interaction matrix (number of visits) for the Piedmont site.

**Table S3.** List of the ant species found in pitfall traps and on EFNs at each site.
