# Supplementary material for: Extrafloral nectar fuels ant life in deserts
Source: AoB Plants. 2014 Nov 7;6:plu068. doi: 10.1093/aobpla/plu068 (PMC4262941; doi:10.1093/aobpla/plu068)
Supplement: Additional Information [file supp_6_plu068_index.html]

Extrafloral nectar fuels ant life in deserts — Additional Information 

# Extrafloral nectar fuels ant life in deserts

## Additional Information

Additional Information

**Files in this Data Supplement:**

- Supplementary File 1 - xls file
- Supplementary File 2 - xls file
- Supplementary File 3 - doc file
