# Supplementary material for: Extrafloral nectar fuels ant life in deserts
Source: AoB Plants. 2014 Nov 7;6:plu068. doi: 10.1093/aobpla/plu068 (PMC4262941; doi:10.1093/aobpla/plu068)
Supplement: Additional Information [file supp_plu068_plu068supp_file3.doc]

List of ant species found in pitfall traps and on EFNs at each site

(a)

| Ant family/ species | Piedmont | Jarillal | on EFNs |
| --- | --- | --- | --- |
| DOLICHODERINAE |  |  |  |
| 1*. Dorymyrmex breviscapis* | X |  | X |
| 2*. Dorymyrmex ebeninus* |  | X |  |
| 3*. Dorymyrmex ensifer* | X | X | X |
| 4*. Dorymyrmex exsanguis* | X | X | X |
| 5*. Dorymyrmex planidens* |  | X | X |
| 6*. Dorymyrmex spurius* | X | X | X |
| 7. *Dorymyrmex wolffhuegeli* | X |  | X |
| 8. *Dorymyrmex* sp.1 | X | X | X |
| 9. *Forelius albiventris* | X | X | X |
| 10. *Forelius chalybaeus* | X | X | X |
| 11. *Forelius rufus* | X |  | X |
| FORMICINAE |  |  |  |
| 12. *Brachymyrmex patagonicus* | X | X | X |
| 13. *Camponotus blandus* | X | X | X |
| 14. *Camponotus mus* | X | X | X |
| 15. *Camponotus punctulatus* | X | X | X |
| 16. *Camponotus substitutus* | X |  | X |
| MYRMICINAE |  |  |  |
| 17. *Acromyrmex lobicornis* ^2^ | X | X |  |
| 18. *Acromyrmex striatus* ^2^ | X | X |  |
| 19. *Cephalotes bruchi* ^*^ | X | X | X |
| 20. *Cephalotes liogaster* ^*^ | X | X | X |
| 21. *Crematogaster quadriformis* | X |  | X |
| 22. *Crematogaster rochai* | X |  | X |
| 23. *Cyphomyrmex rimosus* ^2^ | X |  |  |
| 24. *Kalathomyrmex emeryi* ^2^ | X |  |  |
| 25. *Pheidole bergi* | X | X | X |
| 26. *Pheidole spininodis* ^1^ | X | X |  |
| 27. *Pheidole triconstricta* | X |  | X |
| 28. *Pogonomyrmex cunicularius pencosensis* ^3^ | X |  |  |
| 29. *Pogonomyrmex laticeps* ^1^ | X |  |  |
| 30. *Pogonomyrmex brevibarbis niger* ^1^ |  | X |  |
| 31. *Solenopsis* sp.1 | X | X | X |
| 32. *Solenopsis* sp.2 |  | X | X |
| PSEUDOMYRMYCINAE |  |  |  |
| 33. *Pseudomyrmex denticollis* ^*^ | X | X | X |
| 34. *Pseudomyrmex* sp.1^*^ | X | X | X |
| **Total species** | **30** | **23** | **25** |

Feeding habits of non-EFN consumers: ^1^specialized granivores, ^2^fungus growers and ^3^scavengers. ^*^ Species collected only with hand-sampling.
